# CEO greed and corporate technological innovation: Analyst coverage as an external governance mechanism in China’s A-share market

**DOI:** 10.1371/journal.pone.0337179

**Published:** 2025-11-25

**Authors:** Yuanbo Hu, Ruiyuan Cong, Ran Teng, Baolong Ji

**Affiliations:** 1 School of Digital Business, Zhejiang Dongfang Polytechnic, Wenzhou, China; 2 The School of Business, Macau University of Science and Technology, Macau, China; 3 School of Economics, Bohai University, Jinzhou, China; Brunei University London, UNITED KINGDOM OF GREAT BRITAIN AND NORTHERN IRELAND

## Abstract

As the Fourth Industrial Revolution advances, technological innovation has emerged as a key driver for firms to shape core competitiveness. The corporate governance literature recognizes CEO personality traits as key determinants of a firm’s technological innovation. Drawing on Upper Echelons Theory and Behavioral Agency Theory, this study investigates the effect of CEO greed on corporate technological innovation using a sample of China’s A-share companies listed on the Shanghai and Shenzhen stock exchanges from 2011 to 2023. We find that: (1) CEO greed significantly fosters corporate technological innovation. (2) Analyst coverage strengthens the positive relationship between CEO greed and corporate technological innovation. (3) This effect varies significantly across different firms and industries. (4) CEO greed significantly promotes exploitative (shorter-cycle) innovation, but has no significant effect on exploratory (longer-cycle) innovation.

## 1 Introduction

The Fourth Industrial Revolution, driven by advances in artificial intelligence, the Internet of Things, and blockchain, is reshaping the global economy. As traditional industry boundaries blur and technology replacement cycles shorten, technological innovation has become not merely a means for companies to gain a competitive market advantage but a strategic imperative for their long-term survival and development [1,2]. Within this context, the personal traits, cognitive styles, and values of senior managers are viewed as critical determinants of corporate technological innovation [3]. Among these traits, greed, as an extreme form of self-interest, may drive Chief Executive Officers (CEOs) to maximize personal gain, thereby shaping corporate technological innovation [4,5]. Therefore, this study investigates the relationship between CEO greed and corporate technological innovation, aiming to provide valuable insights and implications for corporate governance.

Innovation drives technological advancement and sustainable development. It is a key pathway for firms to build a distinct competitive advantage [6,7]. Its determinants fall into three main areas. First, external factors are a key driver of corporate technological innovation. Among external environmental factors, the attention and communication from institutional investors can significantly promote green innovation [8,9]. In the new energy sector, government subsidies and corporate green innovation exhibit a U-shaped relationship [10]. Second, internal corporate governance provides the internal foundation and institutional framework for technological innovation. Within these mechanisms, the frequency of board meetings and the proportion of independent directors influence corporate technological innovation [11]. Furthermore, stock options for non-executive employees can stimulate risk-taking incentives [12]. This, in turn, spurs corporate technological innovation. Finally, as key corporate decision-makers, the characteristics of CEOs are a critical factor influencing technological innovation. At the biographical level, a CEO’s professional background shapes corporate technological innovation [13]; for instance, an IT background [14] or a financial background [15] can drive it. At the cognitive level, CEO cognitive traits and values influence corporate technological innovation [16], as exemplified by how a CEO’s environmental awareness [17], overseas experience [18], and individualism [19] can enhance corporate green technological innovation. At the personality level, a CEO’s traits impact corporate technological innovation; for instance, characteristics such as CEO overconfidence [20], narcissism [21], and hubris [22] have been found to foster technological innovation.

Among the many personality traits of a CEO, greed is characterized by its self-interest orientation, and its role in corporate governance and management decision-making research has become increasingly prominent. On the one hand, greedy CEOs might harm corporate interests. Existing research indicates that greedy CEOs may be inclined toward decisions such as taking excessive risks, pursuing blind expansion, and accumulating excessive debt [4,5,23], leading to consequences like reduced corporate social responsibility and shareholder wealth. On the other hand, CEO greed can also exhibit positive effects. Some scholars have found that greed can motivate CEOs to focus on achieving corporate objectives [24] and improve workplace safety [25].

Existing research has extensively explored the impact of CEO greed on outcomes such as corporate social responsibility (CSR) [23], shareholder returns [5], and production efficiency [26]. However, a systematic exploration of the intrinsic link between CEO greed and corporate technological innovation remains a notable gap in the literature. This study, therefore, addresses several key questions: (1) Can CEO greed promote corporate technological innovation? (2) What role does analyst coverage play in this relationship? (3) And what type of innovation do greedy CEOs tend to favor? Grounded in Upper Echelons and Behavioral Agency Theories, this study investigates this relationship using a sample of China’s A-share listed companies from 2011 to 2023.

The marginal contributions of this study are as follows: First, our study contributes to the corporate governance literature by integrating CEO greed and corporate technological innovation within a unified analytical framework grounded in Upper Echelons and Behavioral Agency Theories. Second, by examining the moderating role of analyst coverage, this study provides new empirical evidence on the function of external monitoring mechanisms in shaping corporate governance outcomes. Third, our analysis of the heterogeneous impact of CEO greed on technological innovation across different corporate types and industries offers a foundation for developing more targeted corporate governance strategies. Fourth, our research adds considerable nuance by distinguishing between the types of innovation that greedy CEOs prefer, thereby enriching our findings. Collectively, these findings advance the literature on CEO personality traits and provide valuable insights for corporate governance theory and practice.

## 2 Theoretical framework and research hypothesis

### 2.1 The impact of CEO greed on corporate technological innovation

In a business environment characterized by heightened competition and accelerating technological advancements, innovation is not only the cornerstone of a corporation’s survival but also the core driver of its competitive advantage. As key corporate decision-makers, CEOs’ professional backgrounds, cognitive characteristics, and personality traits profoundly influence corporate technological innovation [15,27,28]. Greed, defined as an excessive desire for wealth, power, or status [29,30], may foster corporate technological innovation by influencing CEO decisions, for instance, by increasing the firm’s risk-taking appetite and adjusting its resource allocation.

According to Upper Echelons Theory, the personality traits of top executives significantly shape their decision-making behavior, which in turn affects corporate strategy and performance [3]. On the one hand, greedy CEOs may possess a greater appetite for risk, leading them to implement research and development (R&D) projects with higher potential returns, thereby fostering corporate technological innovation. Technological innovation projects are inherently characterized by high risks and long cycles [31]. In their pursuit of extraordinary returns, greedy CEOs may be more willing to bear the risk of innovation failure, thus elevating the corporation’s risk tolerance, thereby promoting technological innovation. On the other hand, the extreme self-interest of greedy CEOs may drive them to adopt aggressive financing strategies to support R&D projects. To maximize their personal gain [24], these CEOs might resort to excessive debt or equity issuance to raise substantial capital for R&D initiatives. They expect to see performance improvements from innovation in the short term, which can then justify their high compensation, bonuses, or enhance their personal reputation.

From the perspective of Behavioral Agency Theory, managers are not perfectly rational “economic agents.” Their behavioral motives are complex. They are constrained by cognitive biases, emotional factors, and social influences. This leads them to exhibit behavioral patterns that differ from the predictions of traditional Agency Theory [32,33]. First, a greedy CEO’s excessive pursuit of material wealth may lead them to favor high-stakes R&D projects with high expected returns, thereby driving corporate technological innovation [31]. High-risk R&D endeavors are often associated with high rewards. Tempted by the prospect of extraordinary personal gains, such as large bonuses and compensation packages, greedy CEOs are less likely to opt for conservative strategies. Instead, they will be more inclined to select R&D projects with greater potential returns, thus advancing corporate technological innovation. Second, a greedy CEO’s excessive desire for non-material resources [30], such as reputation, social status, and power, may also foster corporate technological innovation. This pursuit can motivate them to view technological innovation as a critical means to enhance the corporation’s competitiveness and realize their personal ambitions. To build a stronger reputation within the industry and solidify their power base, greedy CEOs will proactively invest resources to drive technological innovation.

Based on the preceding analysis, we propose the following hypothesis:

**H1: CEO greed positively influences corporate technological innovation.**

### 2.2 The moderating role of analyst coverage

As a critical information intermediary and an external governance force in the capital market, analyst coverage may influence corporate technological innovation by improving the corporation’s financing environment and by guiding and constraining managerial decisions. On the one hand, analyst coverage can reduce the level of information asymmetry between the company and its investors, thereby fostering technological innovation. By enhancing information transparency, analyst coverage promotes a better understanding of the company among investors and signals its potential value to the market. This, in turn, attracts investment and alleviates the corporation’s financing constraints [34], providing essential capital support for R&D activities and ultimately promoting technological innovation [35,36]. On the other hand, analyst coverage can intensify the external monitoring pressure on a corporation, which also encourages technological innovation. As an external governance mechanism, financial analysts exert a monitoring function, creating market-based discipline that pressures companies to increase their R&D investments, consequently fostering technological innovation [37,38].

Based on the preceding analysis, we propose the following hypothesis:

**H2: Analyst coverage strengthens the positive relationship between CEO greed and corporate technological innovation.**

**Fig 1:**
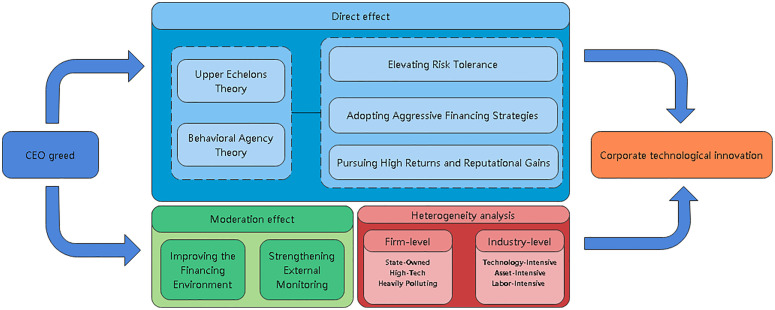
Conceptual framework.

To visually summarize our theoretical framework and the hypotheses proposed, we present the conceptual framework of this study in Fig 1.

## 3 Research design

### 3.1 Sample selection and data sources

This study selects China’s A-share listed companies from 2011 to 2023 as the initial research sample. All data are sourced from the CSMAR and CNRDS databases. We then screen the initial data as follows: (1) we exclude companies in the financial industry; (2) we remove observations with missing data; and (3) we winsorize all continuous variables at the 1st and 99th percentiles. This procedure yields a final sample of 24,766 firm-year observations.

### 3.2 Variable definition

All variables described in this section are measured for each firm-year observation over our sample period from 2011 to 2023. The variable symbols, names, and measurements for all variables are detailed in Table 1.

**Table 1:** Variable definitions.

Variable type	Variable symbol	Variable name	Measurement	Data Source	Access link	Key search words
Dependent variable	*Patent*	Corporate technological innovation	The natural log of (1 + the annual number of patent applications)	CNRDS	https://www.cnrds.com/Home/Login	Patent applications
	*Patent_Award*		The natural log of (1 + the annual number of patents granted)			Patents awarded
Independent variable	*CEO_Greed*	CEO greed	See Section 3.2.1 for a detailed description	CSMAR	https://data.csmar.com/	Executive compensation, Executive age, Executive tenure, CEO duality, Proportion of independent directors, Total assets, Leverage ratio, Sales growth rate, Return on assets (ROA), Stock return
Moderating variable	*Analyst*	The number of analysts covering a firm	The natural log of (1 + the number of analysts covering a firm)			Analyst coverage
	*Report*	The number of analyst reports covering a firm	The natural log of (1 + the number of analyst reports for the same firm)			Analyst report coverage
Control variables	*CEO_gender*	CEO gender	1 if the CEO is female, 0 otherwise			Executive gender
	*CEO_tenure*	CEO tenure	The natural log of the number of months of CEO tenure			Executive tenure
	*CEO_age*	CEO age	Age of the CEO in years			Executive age
	*CEO_Dual*	CEO duality	1 if the CEO is also the Chairman of the Board, 0 otherwise			CEO duality
	*Size*	Firm size	The natural log of the total number of employees at year-end			Number of employees
	*Lev*	Leverage	Total liabilities divided by total assets at year-end			Debt-to-asset ratio
	*BM*	Book-to-market ratio	Book value of equity divided by market value of equity			Book-to-market ratio
	*Cashflow*	Cash flow	Net cash flow from operating activities divided by total assets at year-end			Net operating cash flow, Total assets
	*Cashholdings*	Cash holdings	Cash and cash equivalents divided by total assets at year-end			Cash and cash equivalents, Total assets
	*CapEx*	Capital expenditures	Capital expenditures divided by total assets at the beginning of the year			Capital expenditures, Beginning total assets
	*FIXED*	The proportion of fixed assets	Net property, plant, and equipment (PP&E) divided by total assets at year-end			Fixed asset ratio
	*ATO*	Asset turnover ratio	Total operating revenue divided by average total assets			Total asset turnover
	*Rdpro*	R&D intensity	R&D expenditure divided by total operating revenue			R&D investment ratio

#### 3.2.1 CEO greed.

Greed is an intrinsic personality trait. However, its behavioral manifestation in a corporate setting is most directly observed through an executive’s pursuit of excessive compensation. Therefore, drawing on authoritative literature [5,23,39], this study operationalizes CEO greed using a composite index synthesized via Principal Component Analysis (PCA). This index is derived from three distinct, well-established compensation-based proxies that capture different facets of excessive remuneration: (1) intra-industry pay disparity, reflecting greed relative to external peers; (2) internal pay disparity, reflecting greed relative to internal team members; and (3) CEO overpayment, reflecting compensation that cannot be justified by firm performance or characteristics. This multi-dimensional approach, grounded in multiple authoritative sources, aims to capture the common variance among these indicators, providing a more robust proxy for the underlying latent construct of greed than any single measure alone. Each of these components is detailed below.

The first proxy is the intra-industry pay disparity, measured as the ratio of a corporation’s CEO compensation to the median CEO compensation within the same industry. This indicator assesses the potential excessiveness of a CEO’s pay by comparing it to their industry peers, providing an initial measure of greed.

The second proxy is the internal pay disparity, calculated as the ratio of CEO compensation to the compensation of the second-highest-paid executive. Since CEOs have considerable influence over the compensation of other members of the top management team, a large pay gap is often considered a sign of CEO greed, as it reflects an imbalanced allocation of corporate resources.

The third proxy, CEO overpayment, isolates the portion of CEO compensation that cannot be explained by the determinants of CEO compensation [5,25]. The logic behind this measure is to first model the “normal” or expected level of CEO pay based on a standard set of firm and executive characteristics. The remaining, unexplained portion of compensation—the residual—is then interpreted as excess pay, which serves as our proxy for greed. To implement this, we follow established literature [23,39] and estimate the expected level of CEO compensation using the following regression model:


$$\begin{array}{c} CEO\_Pay_{i,t}=\alpha_{0}+\alpha_{1}CEO\_age_{i,t-1}+\alpha_{2}CEO\_tenure_{i,t-1}+\alpha_{3}CEO\_Dual_{i,t-1}\\ +\alpha_{4}Board\_independence_{i,t-1}+\alpha_{5}Size_{i,t-1}+\alpha_{6}Lev_{i,t-1}\\ +\alpha_{7}Sales\_growth_{i,t-1}+\alpha_{8}ROA_{i,t-1}+\alpha_{9}Stock\_return_{i,t-1}\\ +\sum Year+\sum Firm+\varepsilon_{it} \end{array}$$ (1)

In Model (1), we regress the natural logarithm of total CEO pay (*CEO_Pay*) on a comprehensive set of determinants measured with a one-period lag [25]. These determinants include CEO age (*CEO_age*), CEO tenure (*CEO_tenure*), CEO duality (*CEO_dual*), the percentage of independent directors on the board (*Board_independence*), firm size (*Size*), leverage (*Lev*), sales growth (*Sales_growth*), return on assets (*ROA*), and the 12-month buy-and-hold stock return (*Stock_return*). Year and firm fixed effects are also included to control for unobserved heterogeneity.

From this estimation, we extract the residual (*ε*_*it*_) for each firm-year observation. This residual represents the portion of compensation unexplained by the determinants of CEO compensation in the model. CEO overpayment is then constructed from this residual. Specifically, if the residual is positive (*ε*_*it*_ > 0), it is taken as the measure of CEO overpayment; if the residual is negative or zero (*ε*_*it*_ ≤ 0), CEO overpayment is set to zero. This method ensures an accurate measurement of the excess component of CEO compensation, allowing for a more effective assessment of CEO greed.

Consistent with prior research [5,23], we use Principal Component Analysis (PCA) to combine the three aforementioned compensation-based proxies into a single composite measure representing CEO greed. The PCA yields three factors (eigenvectors) with corresponding estimated eigenvalues of 1.29, 0.95, and 0.76, which are broadly consistent with the findings of O’Sullivan et al. [25]. As the eigenvalue of the first factor is greater than 1 while the other two are less than 1, we adhere to the Kaiser-Guttman selection criterion, which is commonly used in the literature and recommends retaining factors with eigenvalues greater than 1 [40,41]. Consequently, we retain the first principal component with an eigenvalue of 1.29 as our primary measure of CEO greed.

#### 3.2.2 Corporate technological innovation.

Following prior literature [42–44], we measure corporate technological innovation using the annual number of patent applications filed by the listed company. The number of patent applications reflects the output of R&D activities and serves as a direct indicator of a corporation’s innovative efforts. We also use the annual number of patents granted as an alternative measure for robustness checks.

#### 3.2.3 Control variables.

Consistent with prior research [45–48], we include the following control variables: CEO gender (*CEO_gender*), CEO tenure (*CEO_tenure*), CEO age (*CEO_age*), CEO duality (*CEO_Dual*), firm size (*Size*), leverage (*Lev*), book-to-market ratio (*BM*), cash flow (*Cashflow*), cash holdings (*Cashholdings*), capital expenditures (*CapEx*), proportion of fixed assets (*FIXED*), total asset turnover (*ATO*), and R&D intensity (*RDpro*).

#### 3.2.4 Moderating variable.

Consistent with prior studies [49], we measure analyst coverage using two proxies. The primary measure (*Analyst*) is the natural logarithm of one plus the number of securities analysts covering the same listed company. The second measure (*Report*), used for robustness checks, is the natural logarithm of one plus the number of research reports issued for the same company.

### 3.3 Model specification

To test the relationship between CEO greed and corporate technological innovation (H1), we construct the following baseline model:


$$Patent_{it}=\beta_{0}+\beta_{1}CEO\_Greed_{it}+\beta_{2}Controls_{it}+\sum Year+\sum Ind+\varepsilon_{it}$$ (2)

Where the subscript *i* denotes the firm and *t* denotes the year. *Patent* represents the level of corporate technological innovation, *CEO_Greed*_*it*_ is the proxy variable for CEO greed, *Controls*_*it*_ represents a vector of control variables, *Year* and *Ind* are year and industry fixed effects, respectively, and *ε*_*it*_ is the error term.

### 3.4 Sample characteristics and data distribution

This section provides a visual overview of the sample characteristics and the distribution of key variables.

**Fig 2:**
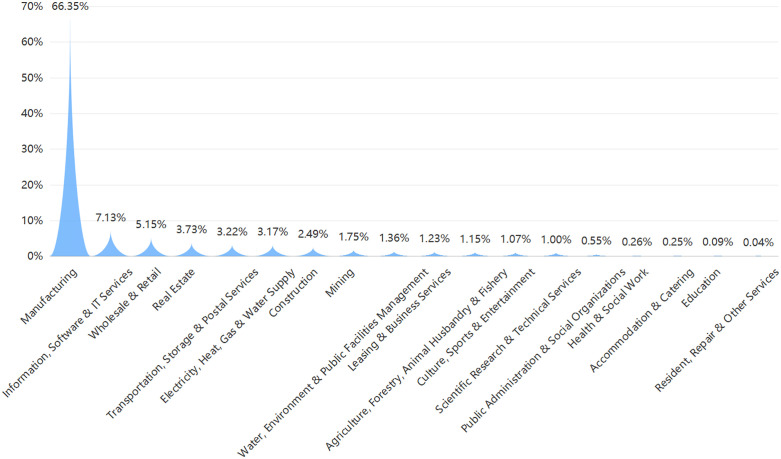
Sample distribution by industry.

First, Fig 2 presents the industry composition of our sample. As shown, the sample is highly concentrated in Manufacturing, which accounts for 66.35% of all firm-year observations, followed by Information, Software & IT Services (7.13%) and Wholesale & Retail (5.15%). All other industries each account for less than 4% of observations, indicating that technology-oriented sectors constitute the bulk of Chinese listed firms engaged in innovation during our study period.

**Fig 3:**
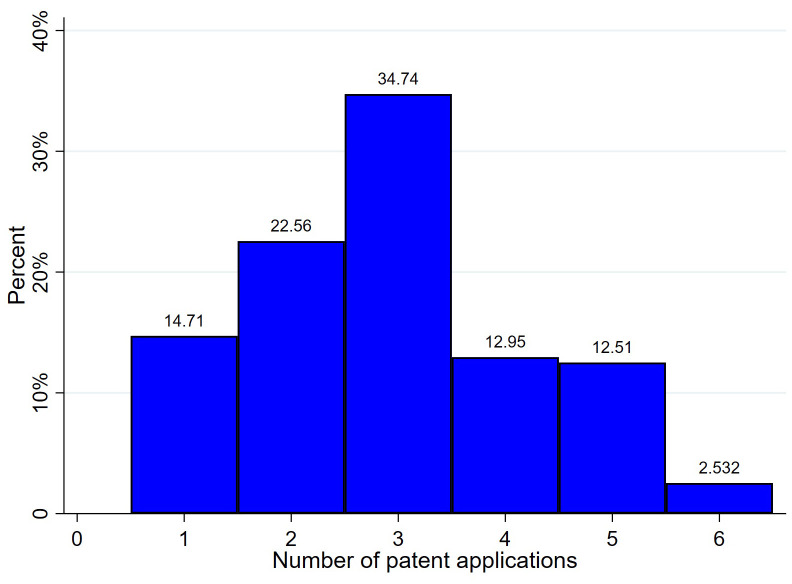
Frequency distribution of patent applications. The x-axis categories denote patent-count bins coded as follows: 0 (code 1), 1–10 (code 2), 11–50 (code 3), 51–100 (code 4), 101–500 (code 5), and >500 (code 6).

Second, Fig 3 illustrates the frequency distribution of the raw number of patent applications across the sample. The chart reveals two key features of the innovation data that are critical for our empirical strategy. First, the data are zero-inflated, with a substantial portion of the sample (14.71%) reporting no patent applications in a given year. Second, the distribution is extremely right-skewed. The majority of observations are concentrated in the lower-end bins—1–10 (22.56%) and 11–50 (34.74%)—while a very small fraction of highly innovative firms (2.53%) accounts for a disproportionately large number of patents (>500). This highly skewed distribution, a common characteristic of patent data, underscores the necessity of the log-transformation (i.e., ln(1 + *Patent*)) used in our subsequent regression analyses to mitigate the influence of outliers and normalize the data.

**Fig 4:**
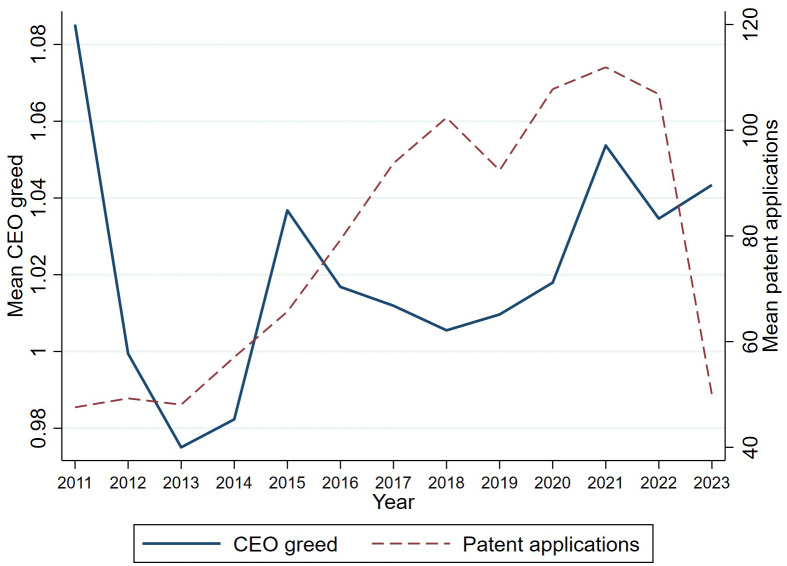
Time trends of mean CEO greed and patent applications (2011–2023).

Finally, Fig 4 plots the time series of the cross-sectional means of CEO greed (left axis) and patent applications (right axis) from 2011 to 2023. CEO greed declined in the aftermath of the 2011 IPO reform but began a steady rebound after 2014, mirroring the upward trajectory of patenting intensity. Both series peak around 2021 before tapering in 2023, consistent with the post-pandemic slowdown. The parallel movement suggests that macro shocks influencing executive incentives may travel hand-in-hand with firms’ innovation appetite, a pattern we investigate more formally in Section 4.

### 3.5 Descriptive statistics

Table 2 presents the descriptive statistics for the main variables used in this study. The core explanatory variable, CEO greed (*CEO_Greed*), exhibits a mean of 1.022 and a standard deviation of 0.973. The variable ranges up to a maximum of 6.634, while its median of 0.720 is considerably lower than the mean. This indicates that the distribution of CEO greed is markedly right-skewed, a common finding in studies on executive compensation [25]. This suggests that while most CEOs in the sample exhibit relatively low levels of greed, a subset of CEOs displays characteristics far exceeding the average. For our measures of technological innovation, the mean values for the number of patent applications (*Patent*) and patent grants (*Patent_Award*) are 2.872 and 2.666, respectively. These variables also show high dispersion, with standard deviations of 1.767 and 1.697 and maximum values of 9.269 and 8.965, respectively. This suggests considerable variation in the intensity and effectiveness of innovation activities among the sample corporations, a typical feature of patent data [42,50–52]. Furthermore, the other variables do not show abnormal extreme values, indicating that the overall variable selection is reasonable.

**Table 2:** Descriptive statistics.

Variable	Obs	Mean	SD	Min	Median	Max	VIF
*Patent*	24766	2.872	1.767	0.000	3.045	9.269	
*Patent_Award*	24766	2.666	1.697	0.000	2.773	8.965	
*CEO_Greed*	24766	1.022	0.973	0.000	0.720	6.634	1.08
*CEO_gender*	24766	0.069	0.254	0.000	0.000	1.000	1.01
*CEO_tenure*	24766	4.907	3.798	0.000	4.167	17.583	1.14
*CEO_age*	24766	50.501	6.674	33.000	51.000	69.000	1.15
*CEO_Dual*	24766	0.275	0.446	0.000	0.000	1.000	1.14
*Size*	24766	7.810	1.198	4.466	7.727	11.187	1.47
*Lev*	24766	0.424	0.192	0.046	0.419	0.866	1.54
*BM*	24766	0.631	0.255	0.032	0.622	1.636	1.43
*Cashflow*	24766	0.105	0.158	−0.535	0.089	0.791	1.15
*Cashholdings*	24766	0.155	0.112	0.010	0.124	0.631	1.33
*CapEx*	24766	0.055	0.055	0.000	0.038	0.351	1.14
*FIXED*	24766	0.209	0.153	0.002	0.180	0.715	1.34
*ATO*	24766	0.648	0.432	0.080	0.553	3.173	1.25
*RDpro*	24766	0.045	0.056	0.000	0.034	1.230	1.28

To ensure the robustness of our regression results, we conducted a multicollinearity diagnostic on the model. In econometrics, multicollinearity can inflate the variance of parameter estimates and reduce the statistical power of the model, potentially leading to erroneous inferences. We use the Variance Inflation Factor (VIF) to assess the degree of collinearity among the variables. Consistent with prior research [53], the VIF values for the core explanatory variable, CEO greed (*CEO_Greed*), and all control variables are below the common threshold of 5, indicating that serious multicollinearity is not a concern for our model.

**Fig 5:**
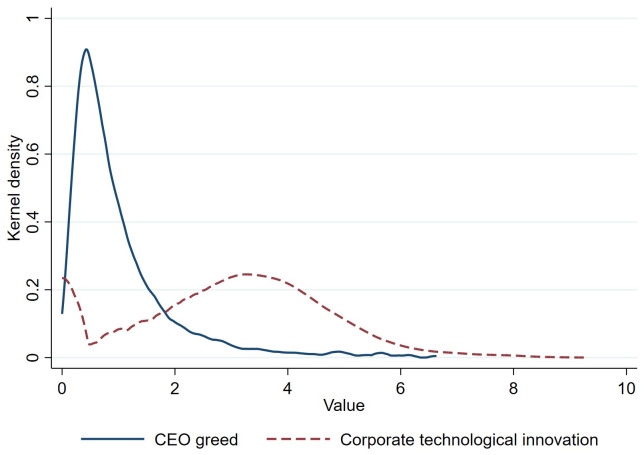
Kernel density plots of CEO greed and corporate technological innovation.

To visually complement the statistics in Table 2, Fig 5 presents the kernel density plots for the distributions of our core explanatory variable, CEO greed, and our dependent variable, corporate technological innovation (log-transformed). Two features stand out: (1) Pronounced right-skewness. Both variables display long right tails, but the patent distribution is far flatter and more dispersed than CEO greed. (2) Different modal regions. CEO greed is tightly clustered near zero, whereas the mode of patent lies between 2 and 4 units.

## 4 Empirical analysis

### 4.1 Baseline regression analysis

**Fig 6:**
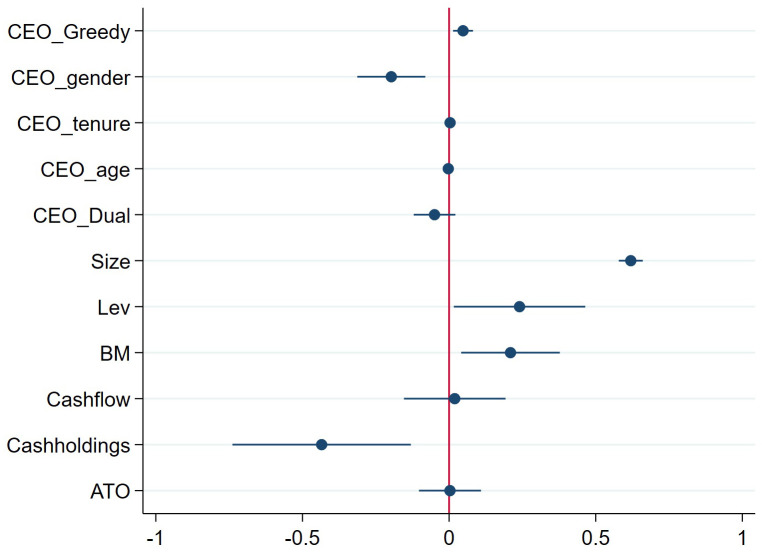
Forest plot of the main effect. This plot visualizes the coefficient on our main variable of interest, *CEO_Greed*. For the sake of visual clarity, the coefficients for the control variables (*CapEx*, *FIXED*, and *RDpro*), which were included in the actual regression models, are omitted from the figure.

This study employs the statistical software Stata 18 to estimate the previously specified econometric model, with the aim of testing the impact of CEO greed on corporate technological innovation. The regression results are presented in Table 3. Specifically, we present a stepwise regression analysis. Column (1) reports the results of a simple regression without control variables or fixed effects. Column (2) shows the results after incorporating industry and year fixed effects, but still without other control variables. Column (3) presents the results with the inclusion of CEO-level control variables, while maintaining the fixed effects. Finally, Column (4) displays the results from the full model, which further includes firm-level control variables in addition to the fixed effects. As shown in Table 3 and Fig 6, the coefficient of *CEO_Greed* is positive and statistically significant at the 1% level across all specifications. Therefore, Hypothesis H1 is supported.

**Table 3:** Baseline regression results.

Variable	(1)	(2)	(3)	(4)
	*Patent*	*Patent*	*Patent*	*Patent*
*CEO_Greed*	0.212^***^	0.216^***^	0.217^***^	0.048^***^
	(7.403)	(9.116)	(9.104)	(2.743)
*CEO_gender*			−0.298^***^	−0.197^***^
			(−4.193)	(−3.330)
*CEO_tenure*			0.007	0.003
			(1.341)	(0.838)
*CEO_age*			0.004	−0.003
			(1.177)	(−1.130)
*CEO_Dual*			−0.214^***^	−0.050
			(−4.765)	(−1.367)
*Size*				0.620^***^
				(29.638)
*Lev*				0.240^**^
				(2.102)
*BM*				0.209^**^
				(2.441)
*Cashflow*				0.019
				(0.218)
*Cashholdings*				−0.434^***^
				(−2.801)
*CapEx*				1.044^***^
				(4.016)
*FIXED*				−1.866^***^
				(−11.891)
*ATO*				0.003
				(0.057)
*RDpro*				3.633^***^
				(8.695)
*_cons*	2.656^***^	2.652^***^	2.518^***^	−1.868^***^
	(69.079)	(86.715)	(17.325)	(−10.118)
*Year*	NO	YES	YES	YES
*Ind*	NO	YES	YES	YES
N	24766	24766	24766	24766
Adj. R^2^	0.014	0.311	0.315	0.490

This table reports the regression results for the impact of CEO greed on corporate technological innovation. Column (1) presents a simple regression without controls or fixed effects. Column (2) adds year and industry fixed effects. Column (3) further includes CEO-level control variables. Column (4) presents the full model with the inclusion of firm-level control. The t-statistics in parentheses are based on standard errors clustered at the firm level. ^*^, ^**^, and ^***^ denote statistical significance at the 10%, 5%, and 1% levels, respectively.

### 4.2 Robustness checks

The results of the robustness checks are presented in Table 4.

**Table 4:** Robustness checks.

Variable	(1)	(2)	(3)	(4)	(5)	(6)
	*Patent_Award*	*Patent*	*Patent*	*Patent*	*Patent*	*Patent*
*CEO_Greed*	0.050^***^		0.046^***^	0.048^***^	0.048^***^	0.051^***^
	(2.957)		(2.630)	(2.802)	(2.755)	(2.844)
*L.CEO_Greed*		0.057^***^				
		(3.119)				
*_cons*	−1.759^***^	−1.731^***^	−1.872^***^	−1.888^***^	−1.868^***^	−1.761^***^
	(−9.855)	(−8.797)	(−10.049)	(−10.212)	(−10.186)	(−8.919)
*Controls*	YES	YES	YES	YES	YES	YES
*Year*	YES	YES	YES	NO	NO	NO
*Ind*	YES	YES	YES	NO	YES	YES
*Ind×Year*	NO	NO	NO	YES	NO	NO
*Pro×Year*	NO	NO	NO	NO	YES	NO
*City×Year*	NO	NO	NO	NO	NO	YES
N	24766	20824	20861	24756	24765	23225
Adj. R^2^	0.495	0.482	0.485	0.495	0.496	0.505

This table presents the results of several robustness checks. Column (1) uses an alternative dependent variable, the number of patents granted (*Patent_Award*). Column (2) addresses potential reverse causality using a lagged specification where all independent and control variables are lagged by one year. Column (3) excludes observations from 2015 and 2020. Column (4) includes industry-year interaction fixed effects to control for time-varying industry factors. Column (5) includes province-year interaction fixed effects for time-varying provincial factors. Column (6) includes city-year interaction fixed effects for time-varying municipal factors. The dependent variable in columns (2) through (6) is the number of patent applications (Patent). The t-statistics in parentheses are based on standard errors clustered at the firm level. ^*^, ^**^, and ^***^ denote statistical significance at the 10%, 5%, and 1% levels, respectively.

#### 4.2.1 Replacing the dependent variable.

Drawing on prior methods for measuring corporate technological innovation [54], we replace our original dependent variable with the number of patent grants (*Patent_Award*) to re-estimate the baseline model. This test serves to verify the robustness of our baseline regression results. As shown in Column (1) of Table 4, the regression coefficient for *CEO_Greed* is 0.05 and remains positive and significant at the 1% level. Furthermore, this coefficient is quantitatively similar to that in the baseline regression, indicating that our baseline findings are robust.

#### 4.2.2 Addressing reverse causality with a lagged variable.

To address the potential reverse causality issue, where firm innovation in period t could influence CEO compensation in the same period, we re-estimate our baseline model using one-year lagged values for both the independent variable (*L.CEO_Greed*) and all control variables. As shown in Column (2) of Table 4, the coefficient of the lagged *CEO_Greed* is 0.057 and remains positive and significant at the 1% level. This result suggests that prior-period CEO greed indeed influences current-period innovation, alleviating concerns about reverse causality and further strengthening our main conclusion.

#### 4.2.3 Altering the sample period.

Given that the 2015 stock market crash in mainland China and the global outbreak of the COVID-19 pandemic in 2020 may have had significant impacts on corporate technological innovation, we re-estimate the regression after excluding the sample years of 2015 and 2020 to mitigate potential biases from these major macroeconomic shocks. This approach is consistent with prior research [55]. The results, presented in Column (3) of Table 4, show that the coefficient of *CEO_Greed* is 0.046 and remains statistically significant at the 1% level. This further suggests that the baseline results are robust.

#### 4.2.4 Controlling for interactive fixed effects.

To account for the possibility that unobserved time-variant factors at the industry, province, and city levels might affect our results, we further control for these interaction fixed effects to test the robustness of our estimates. The results are reported in Columns (4), (5), and (6) of Table 4. The coefficient of *CEO_Greed* remains positive and significant at the 1% level in all these specifications, and its magnitude is largely consistent with the baseline regression. This provides further validation for the robustness of our baseline findings.

#### 4.2.5 Addressing endogeneity concerns.

The results of the analyses addressing endogeneity are presented in Table 5.

**Table 5:** Endogeneity tests.

Variable	(1)	(2)	(3)	(4)
	PSM		2SLS	
	Nearest neighbor matching	Kernel matching	First-stage	Two-stage
	*Patent*	*Patent*	*CEO_Greed*	*Patent*
*CEO_Greed*	0.040^**^	0.042^**^		0.070^***^
	(2.237)	(2.534)		(2.610)
*IV1*			0.700^***^	
			(53.956)	
*IV2*			0.037^***^	
			(2.755)	
*Controls*	YES	YES	YES	YES
*Year*	YES	YES	YES	YES
*Ind*	YES	YES	YES	YES
N	18892	24724	19584	19584
Adj. R^2^	0.501	0.488	0.523	0.281
Kleibergen-Paap rk LM statistic			332.354 [0.000]	
Kleibergen-Paap rk Wald F statistic			1478.836 <19.93>	
Hansen J statistic			[0.3316]	

This table reports the results of endogeneity tests using Propensity Score Matching (PSM) and the Instrumental Variable (IV) methods. For the PSM analysis, firms are assigned to a treatment group if their *CEO_Greed* score is above the sample median, and to a control group otherwise. We employ two common methods, one-to-one nearest neighbor matching and kernel matching, using the baseline control variables as covariates. The main regression is then re-estimated on the matched samples. For the IV regressions, p-values for the relevant diagnostic tests are reported in brackets [ ], and the critical value for the Stock-Yogo weak instrument test at the 10% maximal IV size is in angle brackets <>. The t-statistics in parentheses are based on standard errors clustered at the firm level. ^*^, ^**^, and ^***^ denote statistical significance at the 10%, 5%, and 1% levels, respectively.

(1) **Propensity score matching method**

To mitigate potential endogeneity issues arising from self-selection bias, we employ the Propensity Score Matching (PSM) method. The procedure is as follows. First, we divide the sample into a treatment group and a control group based on the median value of *CEO_Greed*. Second, we apply two commonly used matching algorithms: 1:1 nearest-neighbor matching without replacement and kernel matching. Third, using the same set of control variables from our main analysis as covariates, we match each company in the treatment group with a control company that has similar characteristics. Finally, we re-estimate the regression using the successfully matched sample. As shown in Columns (1) and (2) of Table 5, the regression coefficient for *CEO_Greed* is positive and significant at the 5% level under both the 1:1 nearest-neighbor matching and the kernel matching methods. These results are largely consistent with our baseline findings, indicating that our conclusions are reliable.

(2) **Instrumental variable approach**

To address potential endogeneity issues arising from reverse causality and omitted variables, we employ an instrumental variable (IV) approach, consistent with prior literature [56]. We use the mean value of CEO greed within the same region and year as our first instrumental variable (*IV1*). On the one hand, CEOs within the same region often face similar economic, cultural, and social environments, which may influence their levels of greed. Therefore, a correlation exists between the regional average CEO greed and individual CEO greed, satisfying the relevance condition. On the other hand, the regional average of CEO greed is unlikely to have a direct impact on the technological innovation of a specific corporation in the current period, thus satisfying the exclusion restriction. Additionally, drawing on the work of Acconcia et al. [57], we use the one-year lagged value of the explanatory variable as a second instrumental variable (*IV2*).

Columns (3) and (4) of Table 5 present the regression results from the two-stage least squares (2SLS) estimation. The first-stage results show that the regression coefficients for both *IV1* and *IV2* are positive and significant at the 1% level. Furthermore, the Kleibergen-Paap rk LM statistic significantly rejects the null hypothesis of underidentification, indicating that the model is identified and the instruments are relevant. The Kleibergen-Paap rk Wald F statistic is substantially larger than the Stock-Yogo critical value at the 10% significance level, suggesting that weak instruments are not a concern. The p-value of the Hansen J statistic is greater than 0.1, so we fail to reject the null hypothesis that all instruments are exogenous, thereby confirming the validity of our chosen instruments. In the second-stage regression, the coefficient of *CEO_Greed* remains positive and significant at the 1% level. This result further supports the robustness of our study’s conclusions.

## 5 Test of the moderation effect

Table 6 presents the results of our moderation analysis. As shown in Columns (1) and (2), the coefficients of the interaction terms (*c_CEO_Greed × c_Analyst* and *c_CEO_Greed × c_Report*) are both positive and significant at the 5% level. This indicates that the positive effect of CEO greed on corporate technological innovation is more pronounced when analyst coverage is higher. Thus, Hypothesis H2 is supported.

**Table 6:** Moderation analysis.

Variable	(1)	(2)
	*Patent*	*Patent*
*c_CEO_Greed*	0.021	0.021
	(1.361)	(1.371)
*c_Analyst*	0.190^***^	
	(11.140)	
*c_CEO_Greed×c_Analyst*	0.036^***^	
	(2.865)	
*c_Report*		0.151^***^
		(11.133)
*c_CEO_Greed×c_Report*		0.029^***^
		(2.823)
*_cons*	−1.141^***^	−1.147^***^
	(−5.917)	(−5.958)
*Controls*	YES	YES
*Year*	YES	YES
*Ind*	YES	YES
N	24766	24766
Adj. R^2^	0.501	0.501

This table examines the moderating role of Analyst Coverage in the relationship between CEO greed and corporate technological innovation. *Analyst* is measured as the natural logarithm of one plus the number of financial analysts following the firm. *Report* is measured as the natural logarithm of one plus the number of research reports issued for the firm. To mitigate potential multicollinearity, the independent variable and the moderator were mean-centered prior to creating the interaction term. The t-statistics in parentheses are based on standard errors clustered at the firm level. ^*^, ^**^, and ^***^ denote statistical significance at the 10%, 5%, and 1% levels, respectively.

**Fig 7:**
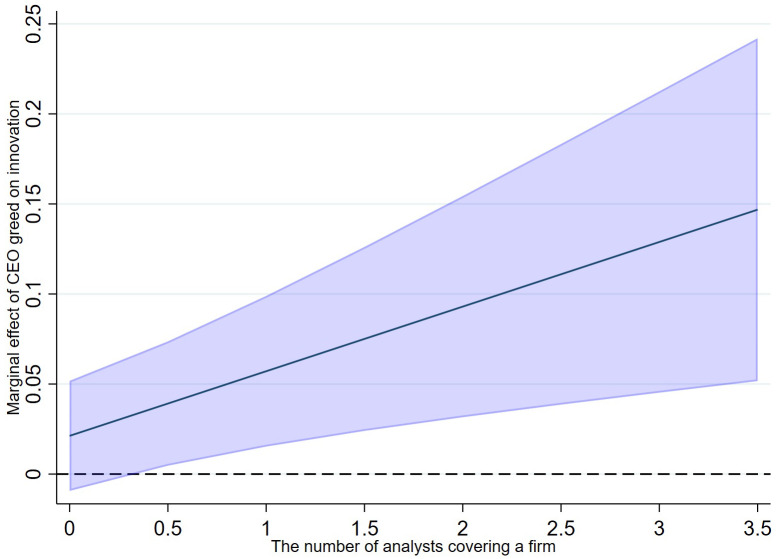
Marginal effect of CEO greed conditional on analyst coverage.

**Fig 8:**
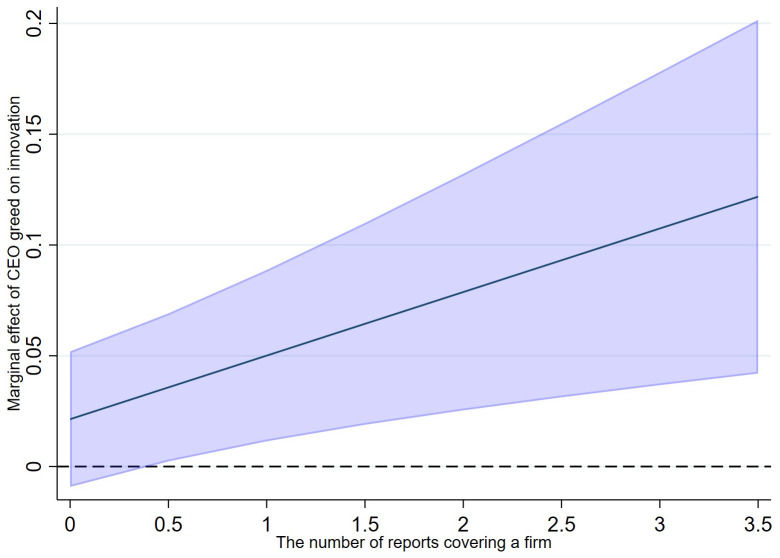
Marginal effect of CEO greed conditional on analyst reports.

To provide a more rigorous and intuitive interpretation of these interaction effects, we first visualize the marginal effects. Fig 7 plots the marginal effect of CEO greed on innovation conditional on the number of analysts covering the firm. The plot shows a positive and upward-sloping line, indicating that the positive effect of CEO greed on innovation is statistically significant and its magnitude increases with the level of analyst coverage. Similarly, Fig 8 shows that the marginal effect of CEO greed also strengthens as the number of analyst reports increases.

**Fig 9:**
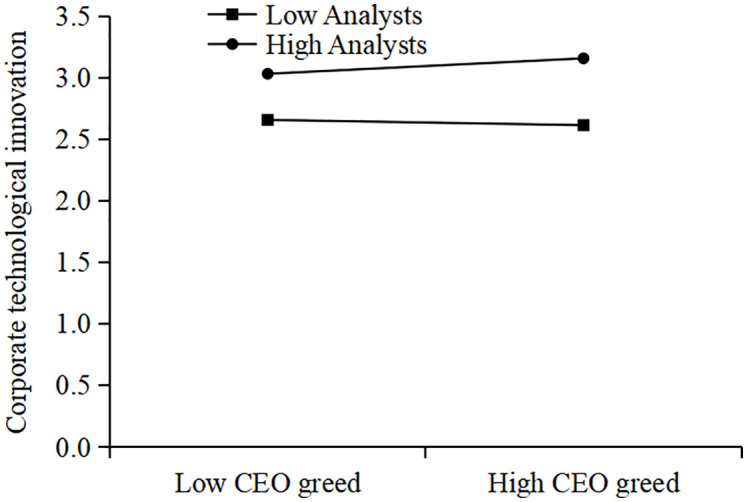
Plot of the interaction between CEO greed and analyst coverage.

**Fig 10:**
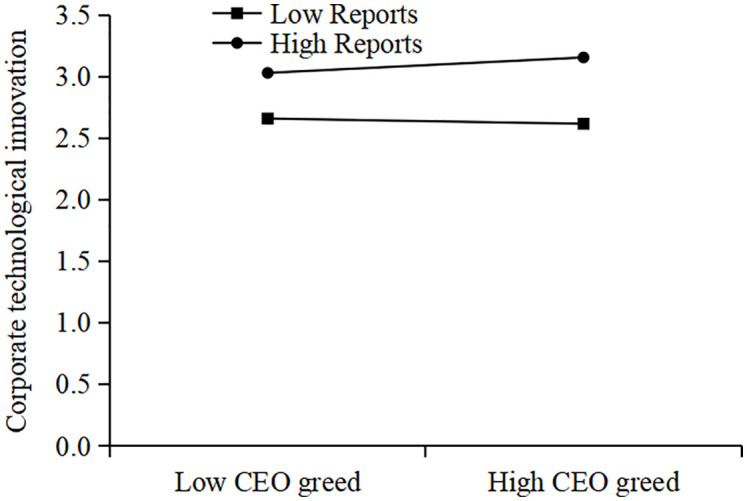
Plot of the interaction between CEO greed and analyst reports.

To further illustrate this moderating relationship from the perspective of predicted outcomes, we also present traditional interaction plots. Fig 9 plots the relationship between CEO greed and innovation at high (mean + 1 SD) and low (mean – 1 SD) levels of analyst coverage, while Fig 10 shows the same for high and low levels of analyst reports. In both figures, the slope for the “high coverage” group is visibly steeper than for the “low coverage” group. Collectively, these visualizations provide strong, consistent support for H2.

## 6 Further analysis

### 6.1 Heterogeneity analysis

The baseline regression confirms a positive relationship between CEO greed and firm innovation. However, this average effect may mask significant variation across firms with different characteristics and operating in different industrial contexts. To explore this, we conduct a series of heterogeneity analyses by splitting our sample based on key firm-level and industry-level characteristics. At the firm level, we examine differences based on ownership structure, technological attributes, and environmental impact profile. At the industry level, we consider heterogeneity across technology-intensive, asset-intensive, and labor-intensive industries. The detailed criteria for each of these subgroup classifications are presented in Table 7.

**Table 7:** Definitions for heterogeneity analysis groupings.

Analysis Type	Group Definition	Classification Criteria	Coding
Firm-level Heterogeneity	State-Owned Firms	The ultimate controller of the firm is identified as a state-owned enterprise, administrative agency, public institution, or central/local government body. If a firm has multiple ultimate controllers, it is classified as state-owned if at least one of them is a state entity, based on the CSMAR Data Manual.	Dummy variable equals 1 if the firm is state-owned, 0 otherwise.
	High-Tech Firms	Based on the 2012 CSRC Industry Classification Guidelines, firms with industry codes of C25–C29, C31–C32, C34–C41, I63–I65, or M73 are defined as high-tech firms [58].	Dummy variable equals 1 if the firm is in a high-tech industry, 0 otherwise.
	Heavily Polluting Firms	Based on the 2012 CSRC Industry Classification Guidelines, firms with industry codes of B06, B07, B08, B09, C17, C19, C22, C25, C26, C28, C29, C30, C31, C32, or D44 are defined as heavily polluting firms [59].	Dummy variable equals 1 if the firm is in a heavily polluting industry, 0 otherwise.
Industry-level Heterogeneity	Technology-Intensive Industries	Based on the 2012 CSRC Industry Classification Guidelines, firms with industry codes of N77, C36, M74, I65, C33, C35, C27, C29, C39, C38, C37, C41, or C40 are defined as being in technology-intensive industries [60].	Dummy variable equals 1 if the firm is in a technology-intensive industry, 0 otherwise.
	Asset-Intensive Industries	Based on the 2012 CSRC Industry Classification Guidelines, firms with industry codes of G56, D44, A04, B11, D45, B07, C22, C31, G55, C30, R86, C28, C26, or C25 are defined as being in asset-intensive industries [61].	Dummy variable equals 1 if the firm is in an asset-intensive industry, 0 otherwise.
	Labor-Intensive Industries	Based on the 2012 CSRC Industry Classification Guidelines, firms with industry codes of A01–A03, A05, B06, B08, B09, C13–C15, C17–C21, C23, C24, C32, C34, D46, E48–E50, F51, F52, G53, G54, G58, G59, I63, I64, K70, L72, M73, M75, N78, P82, R85, R87, or S90 are defined as being in labor-intensive industries [60].	Dummy variable equals 1 if the firm is in a labor-intensive industry, 0 otherwise.

These subgroup classifications based on firm and industry characteristics are not mutually exclusive. A single firm can belong to multiple categories simultaneously. For instance, based on the 2012 CSRC industry codes, some firms in high-tech industries (e.g., “Manufacture of rubber and plastic products,” code C29) are also classified as being in heavily polluting industries. Our analytical approach addresses this by treating each classification as an independent dimension of heterogeneity. Each subsample analysis (e.g., comparing High-Tech vs. Non-High-Tech firms) utilizes the full sample to compare the heterogeneous effect of CEO greed across these subgroups, treating each classification as an independent dimension. This method allows us to isolate the distinct contextual influence of each characteristic on the main relationship.

#### 6.1.1 Firm-level heterogeneity analysis.

To further investigate the differential effects of CEO greed on corporate technological innovation across various firm types, this study conducts a heterogeneity analysis based on firms’ ownership structure [62–64], technological attributes [65,66], and environmental impact characteristics [67,68]. The results are presented in Table 8.

**Table 8:** Firm-level heterogeneity analysis.

Variable	(1)	(2)	(3)	(4)	(5)	(6)
	State-Owned Firms	Non-State-Owned Firms	High-Tech Firms	Non-High-Tech Firms	Heavily Polluting Firms	Non-Heavily Polluting Firms
	*Patent*	*Patent*	*Patent*	*Patent*	*Patent*	*Patent*
*CEO_Greed*	0.065^**^	0.036^*^	0.058^***^	0.040	0.002	0.068^***^
	(2.142)	(1.653)	(2.922)	(1.358)	(0.068)	(3.600)
*_cons*	−2.171^***^	−1.517^***^	−2.168^***^	−1.921^***^	−3.363^***^	−1.851^***^
	(−6.182)	(−6.705)	(−10.076)	(−6.206)	(−8.060)	(−9.005)
*Controls*	YES	YES	YES	YES	YES	YES
*Year*	YES	YES	YES	YES	YES	YES
*Ind*	YES	YES	YES	YES	YES	YES
N	8674	14383	14798	9966	5333	19429
Adj. R^2^	0.597	0.421	0.443	0.468	0.369	0.535

This table explores the impact of CEO greed on corporate technological innovation across different firm classifications. The t-statistics in parentheses are based on standard errors clustered at the firm level. ^*^, ^**^, and ^***^ denote statistical significance at the 10%, 5%, and 1% levels, respectively.

Columns (1) and (2) distinguish firms by ownership structure. We find that the promotional effect of CEO greed on corporate technological innovation is significant in both state-owned and non-state-owned firms. However, the coefficient is larger and more significant in the state-owned firm subsample. A possible explanation is that, compared to their non-state-owned counterparts, state-owned firms often control more monopolistic resources and receive greater policy support from the government [69]. In such an environment, greed is more likely to drive CEOs to pursue personal reputation, career advancement, and salary growth by converting these state-owned advantages into corporate R&D strengths, thereby more effectively fostering technological innovation.

When the sample is divided based on whether a firm is classified as high-tech, the results in columns (3) and (4) show a clear divergence. CEO greed significantly enhances corporate technological innovation only in the high-tech firm group, while the coefficient for the non-high-tech firm group is not significant. This is likely because innovation is the primary engine of growth for high-tech firms, which aligns with the pursuit of high returns characteristic of greedy CEOs [70]. Greedy CEOs in this context may secure substantial rewards through continuous R&D breakthroughs and product iterations, thus stimulating innovation. In contrast, technological iteration in non-high-tech firms is typically slower, and R&D outcomes are less likely to translate into short-term personal performance, which may inhibit the innovative drive of a greedy CEO.

The heterogeneity analysis from an environmental impact perspective, shown in columns (5) and (6), reveals that the positive effect of CEO greed on corporate technological innovation is mainly concentrated in the non-heavily polluting firms subsample; this effect is not significant in the heavily polluting group. This could be because heavily polluting firms face stricter environmental regulatory pressures, compelling greedy CEOs to prioritize the allocation of limited resources toward environmental governance, consequently suppressing corporate technological innovation. Specifically, these firms are subject to tighter environmental constraints, such as emission standards, risks of production suspension, carbon quotas, and pollution discharge fees. Since environmental compliance is rigid and requires immediate action, a greedy CEO, aiming to avoid fines and production disruptions to secure current performance, will tend to allocate finite funds to end-of-pipe treatment and equipment upgrades that ensure rapid compliance. This creates a distinct crowding-out effect on corporate R&D investment [71,72].

#### 6.1.2 Industry-level heterogeneity analysis.

To further dissect the varying impacts of CEO greed on corporate technological innovation across different industries, this study examines heterogeneity based on industry-level factor intensity [73–75]. The regression results are reported in Table 9.

**Table 9:** Industry-level heterogeneity analysis.

Variable	(1)	(2)	(3)	(4)	(5)	(6)
	Technology-Intensive Industries	Non- Technology- Intensive Industries	Asset- Intensive Industries	Non- Asset- Intensive Industries	Labor- Intensive Industries	Non- Labor- Intensive Industries
	*Patent*	*Patent*	*Patent*	*Patent*	*Patent*	*Patent*
*CEO_Greed*	0.069^***^	0.033	−0.004	0.069^***^	0.069^**^	0.040^*^
	(3.062)	(1.358)	(−0.096)	(3.682)	(2.269)	(1.935)
*_cons*	−2.281^***^	−1.982^***^	−3.310^***^	−1.818^***^	−1.679^***^	−2.167^***^
	(−9.778)	(−7.520)	(−6.928)	(−9.009)	(−5.331)	(−10.169)
*Controls*	YES	YES	YES	YES	YES	YES
*Year*	YES	YES	YES	YES	YES	YES
*Ind*	YES	YES	YES	YES	YES	YES
N	11788	12975	4304	20457	8450	16315
Adj. R^2^	0.457	0.459	0.439	0.510	0.478	0.461

This table investigates the impact of CEO greed on different types of corporate technological innovation. The t-statistics in parentheses are based on standard errors clustered at the firm level. ^*^, ^**^, and ^***^ denote statistical significance at the 10%, 5%, and 1% levels, respectively.

First, in the comparison between technology-intensive and non-technology-intensive industries, columns (1) and (2) show that the promotional effect of CEO greed on corporate technological innovation is significantly concentrated in technology-intensive industries, whereas it is insignificant in their non-technology-intensive counterparts. This is likely because the core driver of development in technology-intensive industries is R&D. A successful innovation in this area can rapidly boost a firm’s valuation in the capital market. In contrast, non-technology-intensive industries feature slower technological upgrading and limited patentable space, with revenue structures that rely more on scale expansion, channel investment, and cost control. To pursue high returns in these industries, a greedy CEO is more likely to invest capital, time, and energy into expanding scale, broadening marketing channels, and improving daily management rather than engaging in technological innovation. This finding is consistent with the aforementioned results for high-tech firms.

Second, when firms are categorized by asset intensity, the results in columns (3) and (4) indicate that the positive influence of CEO greed on corporate technological innovation exists primarily in non-asset-intensive industries, while this effect is not significant in asset-intensive industries. This may be because non-asset-intensive industries, which are centered on knowledge capital such as software, design, and consulting, do not rely on large-scale fixed asset investments, complex production line renovations, or long-cycle R&D. Their marginal investment is lower and the outcome validation period is relatively short, which may align with a greedy CEO’s focus on maximizing short-term benefits. In asset-intensive industries, however, corporate technological innovation often faces greater difficulty and longer timelines [76]. As these firms depend on substantial fixed assets like large-scale production equipment and complex industrial processes, any technological change must be implemented without disrupting the existing production system, which significantly increases the difficulty of innovation. Moreover, innovation in these industries typically requires substantial upfront investment in R&D, equipment upgrades, and employee training, with returns that are not immediately visible. In this scenario, a greedy CEO might avoid innovation risks and instead focus on short-term financial gains to maximize personal benefits, for instance, by increasing production scale or expanding market share rather than committing resources to high-risk, long-term technological innovation.

Finally, the comparative analysis between labor-intensive and non-labor-intensive industries in columns (5) and (6) shows that CEO greed significantly promotes corporate technological innovation in both types of industries. However, the regression coefficient for CEO greed is larger and more significant in labor-intensive industries. A potential reason is that technological upgrading in labor-intensive industries often involves process improvements and minor equipment innovations, which require relatively limited investment and offer shorter payback periods [77]. This again aligns with a greedy CEO’s objective of maximizing short-term returns. Therefore, while CEO greed fosters innovation in both settings, its profit-seeking nature may be more pronounced in labor-intensive industries, leading to more frequent implementation of innovative decisions and, consequently, a stronger promotion of corporate technological innovation.

### 6.2 Analysis of corporate technological innovation types

This section further delineates the relationship between CEO greed and the type of corporate technological innovation by classifying it into exploitative innovation (*Exploitative_Innovation*) and exploratory innovation (*Exploratory_Innovation*) [78]. According to organizational learning theory, exploitative innovation focuses on refining existing technologies and processes to enhance efficiency, characterized by relatively low risk and a short return horizon. In contrast, exploratory innovation involves developing entirely new knowledge, technologies, and markets, which entails high uncertainty and a long payback period.

The results presented in Table 10 reveal a differentiated impact of CEO greed on these two types of innovation. Specifically, column (1) shows that CEO greed significantly promotes the firm’s exploitative innovation output. However, its effect on exploratory innovation, as shown in column (2), is not statistically significant. This raises a crucial question: why might a greedy CEO favor exploitative over exploratory innovation? We posit that this differential impact stems from two underlying reasons.

**Table 10:** Analysis of a firm’s choice of innovation type.

Variable	(1)	(2)
	*Exploitative_innovation*	*Exploratory_innovation*
*CEO_Greed*	0.045**	−0.000
	(2.351)	(−0.038)
*_cons*	−2.617***	−0.708***
	(−11.896)	(−6.245)
*Controls*	YES	YES
*Year*	YES	YES
*Ind*	YES	YES
N	19343	19343
Adj R^2^	0.540	0.312

This table investigates the impact of CEO greed on different types of corporate technological innovation. Following Guan and Liu [82], we distinguish between exploitative and exploratory innovation based on the dynamic changes in a firm’s patent classifications. The methodology involves comparing the four-digit International Patent Classification (IPC) codes of a new patent against the firm’s technological portfolio from the preceding five years. Specifically, an innovation is classified as exploitative if all of its four-digit IPC codes fall within the technology classes the firm has previously utilized in the past five years. Conversely, an innovation is classified as exploratory if it contains at least one new four-digit IPC code that was not part of the firm’s patent portfolio in the preceding five years. The t-statistics in parentheses are based on standard errors clustered at the firm level. ^*^, ^**^, and ^***^ denote statistical significance at the 10%, 5%, and 1% levels, respectively.

First, the two innovation activities possess distinct risk-return profiles and payback periods. Exploratory innovation is a high-investment, high-risk, and long-cycle activity, with its future commercial success fraught with substantial uncertainty [78]. Conversely, exploitative innovation, which centers on optimizing existing capabilities, is less risky and yields more predictable outcomes. A greedy CEO, who prioritizes personal compensation and career prospects, seeks to maximize immediate personal gains. Such an executive may therefore actively shun the high failure risk and delayed returns associated with exploratory innovation, opting instead for exploitative innovation projects that can be quickly converted into personal performance metrics and financial rewards [79].

Second, the design of modern corporate compensation contracts may exacerbate the myopic behavior of a greedy CEO. In publicly listed companies, executive compensation schemes are often closely tied to short-term performance metrics, such as annual or quarterly financial reports [80]. This structure can incentivize a greedy CEO to prioritize activities that rapidly improve current financial performance. Exploratory innovation projects not only fail to contribute to profits in the short term but can also depress current performance due to their substantial R&D expenditures. This runs counter to the objective of a greedy CEO focused on maximizing personal short-term remuneration [81]. Consequently, a greedy CEO is more likely to allocate resources to exploitative innovation projects that can deliver a swift boost to short-term financial results.

## 7 Conclusions and implications

### 7.1 Conclusions

As the Fourth Industrial Revolution advances, technological innovation has emerged as a key driver for firms to shape core competitiveness. The personality traits of senior executives, particularly greed, can play a significant role in shaping strategic decisions related to innovation. A deeper understanding of the impact of CEO greed on corporate technological innovation is therefore essential for improving corporate governance mechanisms and stimulating a firm’s innovative capacity. Ultimately, such insights can help firms maintain a competitive advantage in a complex and rapidly changing market environment. Accordingly, drawing on Upper Echelons Theory and Behavioral Agency Theory, this study investigates the effect of CEO greed on corporate technological innovation using a sample of China’s A-share companies listed on the Shanghai and Shenzhen stock exchanges from 2011 to 2023.

This study yields several key findings. First, our results indicate that CEO greed significantly promotes corporate technological innovation. This finding directly corroborates the core tenet of Upper Echelons Theory, which posits that the personal traits of executives are key drivers of corporate technological innovation. The high-risk appetite and desire for excess returns associated with greedy CEOs make them more inclined to champion technology projects characterized by high uncertainty but substantial potential rewards, providing an internal impetus for innovation. This conclusion aligns with Behavioral Agency Theory. It suggests that greedy CEOs’ decisions are not entirely rational. Instead, they are influenced by desires for personal wealth, reputation, and power. To maximize their personal interests, greedy CEOs will proactively advance corporate technological innovation.

Second, our mechanism analysis reveals that analyst coverage positively moderates the relationship between CEO greed and corporate technological innovation. The scrutiny from analysts not only reduces information asymmetry and improves the financing environment for innovation activities but also creates a market-based disciplinary force. This pressure drives greedy CEOs to enhance long-term company value by fostering corporate technological innovation.

Third, the heterogeneity analysis demonstrates that the promotional effect of CEO greed on technological innovation is more pronounced in state-owned firms, high-technology firms, and non-heavily polluting firms. At the industry level, this effect is stronger in technology-intensive, non-capital-intensive, and labor-intensive sectors. For instance, in heavily polluting firms, the positive effect of CEO greed is not significant. This suggests that when faced with more rigid short-term constraints, such as environmental compliance, greedy CEOs allocate resources to avoiding penalties rather than to risky R&D investments, creating a crowding-out effect on corporate technological innovation.

Fourth, we find that CEO greed significantly fosters shorter-cycle exploitative innovation but has no significant impact on long-cycle exploratory innovation. This divergence can be attributed to two factors. On the one hand, the two types of innovation differ markedly in risk, duration, and the predictability of returns; to maximize their immediate personal gains, greedy CEOs prefer exploitative innovation, which offers lower risks and faster payoffs. On the other hand, compensation contracts in modern corporations are often tightly linked to short-term financial metrics. This structure may incentivize greedy CEOs to favor exploitative innovation to meet short-term performance targets, thereby maximizing their current salary and bonuses.

As the strategic importance of corporate technological innovation in global competition grows, CEO greed, as a critical personality trait of senior executives, plays a positive role in promoting this innovation. A systematic analysis of its impact and underlying mechanisms provides a vital evidence base for driving innovation through effective corporate governance.

### 7.2 Implications

Based on these conclusions, we propose the following managerial implications to guide CEO greed appropriately, foster corporate technological innovation, thereby strengthening the firm’s core competitiveness.

First, it is imperative to construct more refined corporate governance mechanisms to channel CEO greed constructively. Given that CEO greed positively stimulates innovation, particularly exploitative innovation, governance should focus on guidance and regulation rather than complete suppression of CEO’s personal motives. The board of directors—especially the compensation and audit committees—should design more balanced and long-term incentive and restraint systems. These systems must move beyond excessively short-term performance metrics to incorporate innovation quality, particularly progress in exploratory innovation, and long-term sustainability goals into executive compensation packages. Concurrently, the supervisory function and professional competence of independent directors must be strengthened to ensure they can effectively identify and counterbalance CEO decisions that might harm the firm’s long-term interests due to excessive greed. This is crucial in the selection and resource allocation of innovation projects to prevent an overemphasis on short-term, visible exploitative innovations at the expense of strategic exploratory initiatives essential for the firm’s future.

Second, the capital market’s information environment should be optimized to strengthen the constraining effect of market supervision on CEO behavior. The finding that analyst coverage amplifies the positive impact of CEO greed on innovation highlights the critical role of a transparent and efficient capital market in resource allocation and incentive alignment. Regulatory bodies should continually enhance information disclosure systems to improve the transparency and comparability of listed firms’ data, while encouraging and standardizing the information-gathering and dissemination functions of intermediaries like analysts and rating agencies. By increasing market transparency and oversight, a dual objective can be achieved: it allows CEOs genuinely committed to innovation and their achievements to gain due market recognition and resource support, while also making it easier for the market to identify and discipline speculative or superficial innovation activities driven solely by personal greed. This ultimately guides CEOs to align their personal ambitions more closely with the creation of value through corporate technological innovation.

Third, policymaking and firm practices must fully consider contextual factors by implementing differentiated innovation guidance strategies. Our research reveals that the innovation effect of CEO greed differs significantly across firms with varying ownership structures, technological attributes, and environmental profiles, as well as across industries with different factor intensities. This implies that undifferentiated governance models or incentive policies are likely to be ineffective. For instance, in state-owned enterprises, while acknowledging that their institutional advantages might amplify a CEO’s drive for innovation, it is crucial to guard against the risks of over-investment or deviation from core business activities. For high-tech firms and in technology-intensive industries, policies should encourage calculated risk-taking while improving risk-sharing mechanisms. Conversely, in heavily polluting firms or industries, relying on CEO greed alone may not effectively drive necessary transformations like green innovation. In such contexts, sustainable innovation is better spurred by external regulatory pressures, targeted policy subsidies, and leaders who possess environmental consciousness and a long-term vision.

Fourth, firms must proactively manage their innovation portfolios to guard against the strategic imbalance that CEO greed may induce. The research clearly indicates that CEO greed tends to drive exploitative rather than exploratory innovation. This can lead a firm into a competency trap, where it becomes overly reliant on existing advantages while neglecting future growth potential, ultimately damaging its long-term competitiveness and sustainability. Therefore, the board and senior management must consciously balance investments in exploitative and exploratory innovation. This requires establishing dedicated organizational units, secure budgets, and specific evaluation systems to support exploratory projects, fostering a tolerance for failure in the exploratory process, and promoting a firm culture that encourages challenging the status quo and pursuing future-oriented innovation. Investors, particularly institutional investors focused on long-term value and sustainability, should also scrutinize the balance of a firm’s innovation portfolio during their engagement, raising red flags for firms that exhibit an excessive bias toward short-term exploitative activities.

## 8 Limitations and future research

This study, while contributing to the literature, is subject to several limitations that offer promising avenues for future research.

First, our study relies on the number of patent applications as the primary proxy for corporate technological innovation. We acknowledge that patents represent a quantifiable but incomplete measure of a firm’s innovative output. This metric may not fully capture non-patentable forms of innovation, such as novel business models, organizational process improvements, or service innovations, nor does it inherently differentiate between high-impact and incremental patents. Despite this limitation, the use of patent data is a widely accepted practice in innovation research, providing an objective and comparable measure of innovative activity that aligns with numerous prior studies. Future research could extend our work by incorporating alternative or complementary metrics, such as R&D expenditure, new product announcements, or citation-weighted patent quality, to provide a more holistic view of the innovation landscape.

Second, our empirical analysis is based exclusively on data from China’s A-share listed companies, which may constrain the generalizability of our findings to other institutional contexts. China’s unique economic environment—characterized by the significant influence of state-owned enterprises, a developing capital market, and distinct cultural norms—undoubtedly shapes the manifestation and consequences of CEO greed. Therefore, caution should be exercised when extrapolating our conclusions to developed markets like the United States or Europe, where governance structures and executive incentive systems differ substantially. Nevertheless, as the world’s second-largest economy, China provides a critically important and compelling setting for studying corporate governance and innovation. We call for future cross-country comparative studies to investigate whether the relationships observed in our research hold in different institutional and cultural settings.

Third, to operationalize the challenging construct of CEO greed, this study adopts a scientifically robust, multi-dimensional approach. Grounded in authoritative prior literature [5,23,39], we employ a composite index derived from three distinct compensation-based proxies, synthesized using Principal Component Analysis (PCA). This method provides a valuable and replicable means of capturing the behavioral manifestations of this intrinsic personality trait. We acknowledge, however, that our compensation-based proxy could, to some extent, also reflect other factors, such as efficient contracting outcomes or specific board-level governance policies. Future research might therefore build upon our findings by employing more direct psychological assessments to further disentangle these complex motivations.

## Supporting information

S1 Text **S1 File.** Data. dta. **S2 File.** Code. do.
(RAR)

## References

[1] Guo F, Yang C, Feng S. The Impact of Digital Economy on Green Technology Innovation and Its Mechanism: Evidence from 274 Cities in China. Emerging Markets Finance and Trade. 2024;60(9):1971–85. doi: 10.1080/1540496x.2023.2297925

[2] Griffin D, Li K, Xu T. Board Gender Diversity and Corporate Innovation: International Evidence. J Financ Quant Anal. 2020;56(1):123–54. doi: 10.1017/s002210901900098x

[3] Hambrick DC, Mason PA. Upper Echelons: The Organization as a Reflection of Its Top Managers. The Academy of Management Review. 1984;9(2):193. doi: 10.2307/258434

[4] Jebran K, Chen S, Cai W. Excess of everything is bad: CEO greed and corporate policies. Rev Quant Finan Acc. 2022;59(4):1577–607. doi: 10.1007/s11156-022-01083-7

[5] Takacs Haynes K, Campbell JT, Hitt MA. When More Is Not Enough. Journal of Management. 2016;43(2):555–84. doi: 10.1177/0149206314535444

[6] Zhu L, Qi Z, Wang C, Liu L, Li T, Ma H. The Impact of Executive External Pay Disparity on Enterprise Innovation: Evidence from Mixed-Ownership Enterprises in China. Emerging Markets Finance and Trade. 2024;60(13):3061–83. doi: 10.1080/1540496x.2024.2333427

[7] Flammer C, Hong B, Minor D. Corporate governance and the rise of integrating corporate social responsibility criteria in executive compensation: Effectiveness and implications for firm outcomes. Strategic Management Journal. 2019;40(7):1097–122. doi: 10.1002/smj.3018

[8] Lv Q, Li X, Sun Y, Han Y. Institutional Investors’ Green Activism and Corporate Green Innovation: Based on the Behind-Scene Communications. Emerging Markets Finance and Trade. 2024;60(14):3284–307. doi: 10.1080/1540496x.2024.2345192

[9] Jiang L, Bai Y. Strategic or substantive innovation? -The impact of institutional investors’ site visits on green innovation evidence from China. Technology in Society. 2022;68:101904. doi: 10.1016/j.techsoc.2022.101904

[10] Xia L, Gao S, Wei J, Ding Q. Government subsidy and corporate green innovation - Does board governance play a role?. Energy Policy. 2022;161:112720. doi: 10.1016/j.enpol.2021.112720

[11] Sierra-Morán J, Cabeza-García L, González-Álvarez N, Botella J. The board of directors and firm innovation: A meta-analytical review. BRQ Business Research Quarterly. 2024;27(2):182–207. doi: 10.1177/23409444211039856

[12] Chang X, Fu K, Low A, Zhang W. Non-executive employee stock options and corporate innovation. Journal of Financial Economics. 2015;115(1):168–88. doi: 10.1016/j.jfineco.2014.09.002

[13] Li H, Yang X, Meng M. Research on the relationship between CEO career variety, digital knowledge base extension, and digital transformation in the context of digital merger and acquisition: The case of China’s new generation of information technology firms. PLoS One. 2024;19(3):e0297044. doi: 10.1371/journal.pone.0297044 38478525 PMC10936827

[14] Shen D, Kim Y. Can Information Technology Background of CEO Enhance Green Innovation? Evidence From China. The Institute Manag Econ Research. 2024;15(4):1–24. doi: 10.32599/apjb.15.4.202412.1

[15] García-Pérez-de-Lema D, Ruiz-Palomo D, Diéguez-Soto J. Analysing the roles of CEO’s financial literacy and financial constraints on Spanish SMEs technological innovation. Technology in Society. 2021;64:101519. doi: 10.1016/j.techsoc.2020.101519

[16] Custódio C, Ferreira MA, Matos P. Do General Managerial Skills Spur Innovation?. Management Science. 2019;65(2):459–76. doi: 10.1287/mnsc.2017.2828

[17] Huang Q, Chen X, Zhou M, Zhang X, Duan L. How Does CEO’s Environmental Awareness Affect Technological Innovation?. Int J Environ Res Public Health. 2019;16(2):261. doi: 10.3390/ijerph16020261 30658493 PMC6352152

[18] Quan X, Ke Y, Qian Y, Zhang Y. CEO Foreign Experience and Green Innovation: Evidence from China. J Bus Ethics. 2023;182(2):535–57. doi: 10.1007/s10551-021-04977-z

[19] Gao L, Han J, Pan Z, Zhang H. Individualistic CEO and corporate innovation: Evidence from U.S. frontier culture. Research Policy. 2023;52(9):104852. doi: 10.1016/j.respol.2023.104852

[20] Li Z, Zhang Y. CEO Overconfidence and Corporate Innovation Outcomes: Evidence From China. Front Psychol. 2022;13:760102. doi: 10.3389/fpsyg.2022.760102 35197895 PMC8858794

[21] Shan B, Liu X, Chen B, Ma J. CEO narcissism and corporate performance in China. China Economic Review. 2023;79:101970. doi: 10.1016/j.chieco.2023.101970

[22] Arena C, Michelon G, Trojanowski G. Big Egos Can Be Green: A Study of CEO Hubris and Environmental Innovation. British J of Management. 2018;29(2):316–36. doi: 10.1111/1467-8551.12250

[23] Sajko M, Boone C, Buyl T. CEO Greed, Corporate Social Responsibility, and Organizational Resilience to Systemic Shocks. Journal of Management. 2021;47(4):957–92. doi: 10.1177/0149206320902528

[24] Haynes KT, Josefy M, Hitt MA. Tipping Point. Journal of Leadership & Organizational Studies. 2015;22(3):265–79. doi: 10.1177/1548051815585171

[25] O’Sullivan D, Zolotoy L, Veeraraghavan M, Overbeck JR. Are Employees Safer When the CEO Looks Greedy?. J Bus Ethics. 2024;198(3):655–73. doi: 10.1007/s10551-024-05820-x

[26] Krekels G, Pandelaere M. Dispositional greed. Personality and Individual Differences. 2015;74:225–30. doi: 10.1016/j.paid.2014.10.036

[27] Gao Y, Tang Y, Zhang J. CEO financial background, managerial ownership, and corporate innovation: Insights from imprinting theory. Front Psychol. 2023;14:1126853. doi: 10.3389/fpsyg.2023.1126853 36865362 PMC9971491

[28] Wang C, Hu Y, Zhang J, Miao C. CEO Media Exposure and Green Technological Innovation Decision: Evidence from Chinese Polluting Firms. Mathematical Problems in Engineering. 2020;2020:1–14. doi: 10.1155/2020/8271621

[29] Seuntjens TG, Zeelenberg M, Breugelmans SM, van de Ven N. Defining greed. Br J Psychol. 2015;106(3):505–25. doi: 10.1111/bjop.12100 25315060

[30] Lambie GW, Haugen JS. Understanding greed as a unified construct. Personality and Individual Differences. 2019;141:31–9. doi: 10.1016/j.paid.2018.12.011

[31] Hall BH, Lerner J. The Financing of R&D and Innovation. Handbook of the Economics of Innovation. Elsevier. 2010. 609–39. doi: 10.1016/s0169-7218(10)01014-2

[32] Pepper A, Gore J. Behavioral Agency Theory. Journal of Management. 2015;41(4):1045–68. doi: 10.1177/0149206312461054

[33] Wiseman RM, Gomez-Mejia LR. A Behavioral Agency Model of Managerial Risk Taking. The Academy of Management Review. 1998;23(1):133. doi: 10.2307/259103

[34] Zhao J, Sun X. To what extent does it deviate from substantive green innovation: the effect of capital market opening on enterprises’ pseudo-green innovation. Applied Economics. 2025;:1–14. doi: 10.1080/00036846.2024.2448612

[35] Li N, Li C, Yuan R, Khan MA, Sun X, Khaliq N. Investor Attention and Corporate Innovation Performance: Evidence from Web Search Volume Index of Chinese Listed Companies. Mathematics. 2021;9(9):930. doi: 10.3390/math9090930

[36] He F, Yan Y, Hao J, Wu J (George). Retail investor attention and corporate green innovation: Evidence from China. Energy Economics. 2022;115:106308. doi: 10.1016/j.eneco.2022.106308

[37] Hu S, Dong W, Huang Y. Analysts’ Green Coverage and Corporate Green Innovation in China: The Moderating Effect of Corporate Environmental Information Disclosure. Sustainability. 2023;15(7):5637. doi: 10.3390/su15075637

[38] Wang KT, Liu S, Luo G. Analyst Coverage and Corporate Innovation: Evidence from Exogenous Changes in Analyst Coverage. Abacus. 2025;61(3):657–710. doi: 10.1111/abac.12360

[39] Rehman SU, Hamdan YH. CEO Greed, Corporate Governance, and CSR Performance: Asian Evidence. Administrative Sciences. 2023;13(5):124. doi: 10.3390/admsci13050124

[40] Brown TE, Davidsson P, Wiklund J. An operationalization of Stevenson’s conceptualization of entrepreneurship as opportunity‐based firm behavior. Strategic Management Journal. 2001;22(10):953–68. doi: 10.1002/smj.190

[41] Mutschmann M, Hasso T, Pelster M. Dark Triad Managerial Personality and Financial Reporting Manipulation. J Bus Ethics. 2022;181(3):763–88. doi: 10.1007/s10551-021-04959-1

[42] Fan S, Luo Y, Dou H, Han W. Enterprise Annuity and Firm Innovation: Evidence from China. Emerging Markets Finance and Trade. 2025;61(6):1479–507. doi: 10.1080/1540496x.2024.2359584

[43] Guedhami O, Pan Y, Saadi S, Zhao D. Do environmental penalties matter to corporate innovation?. Energy Economics. 2025;141:108064. doi: 10.1016/j.eneco.2024.108064

[44] Kang M, Li Y, Zhao Z, Zheng M, Wu H. Does Human Capital Homogeneously Improve the Corporate Innovation: Evidence from China’s Higher Education Expansion in the Late 1990s. Sustainability. 2022;14(19):12352. doi: 10.3390/su141912352

[45] Bradley D, Mao CX, Zhang C. Does Analyst Coverage Affect Workplace Safety?. Management Science. 2022;68(5):3464–87. doi: 10.1287/mnsc.2021.4093

[46] Cohn JB, Wardlaw MI. Financing Constraints and Workplace Safety. The Journal of Finance. 2016;71(5):2017–58. doi: 10.1111/jofi.12430

[47] Qian C, Crilly D, Lin Y, Zhang K, Zhang R. Short-Selling Pressure and Workplace Safety: Curbing Short-Termism Through Stakeholder Interdependencies. Organization Science. 2023;34(1):358–79. doi: 10.1287/orsc.2022.1576

[48] Shi W, Xia C, Meyer-Doyle P. Institutional Investor Activism and Employee Safety: The Role of Activist and Board Political Ideology. Organization Science. 2022;33(6):2404–20. doi: 10.1287/orsc.2021.1542

[49] Dou W, Zhang H, Miao B, Wang B. How do analyst attention and green credit promote corporate green innovation?. International Review of Economics & Finance. 2025;99:104037. doi: 10.1016/j.iref.2025.104037

[50] Lin L, Zheng Y, Tu Y. The new environmental protection law, ESG investment, and corporate innovation performance. International Review of Economics & Finance. 2025;98:103916. doi: 10.1016/j.iref.2025.103916

[51] Feng R, Ma L, Wu D. ESG performance and corporate innovation under the moderating effect of firm size. International Review of Economics & Finance. 2025;97:103774. doi: 10.1016/j.iref.2024.103774

[52] Qiao Y, Li X, Hu J. From digital to innovative: How does digital transformation affect corporate innovation?. Economics Letters. 2025;247:112210. doi: 10.1016/j.econlet.2025.112210

[53] O’brien RM. A Caution Regarding Rules of Thumb for Variance Inflation Factors. Qual Quant. 2007;41(5):673–90. doi: 10.1007/s11135-006-9018-6

[54] Yang X, Gu X, Yang X. Firm age and loan financing with patents as collateral of Chinese startups: the roles of innovations and experience. Economics of Innovation and New Technology. 2023;32(3):343–69. doi: 10.1080/10438599.2021.1916486

[55] Chen F, Zhu L, Zhang H, Li Y. Innovation-driven cities: Reconciling economic growth and ecological sustainability. Sustainable Cities and Society. 2025;121:106230. doi: 10.1016/j.scs.2025.106230

[56] Wang H, Yang L, Feng Y. How does digital economy affect the industry chain resilience in China?. Humanit Soc Sci Commun. 2024;11(1). doi: 10.1057/s41599-024-04077-z

[57] Acconcia A, Corsetti G, Simonelli S. Mafia and Public Spending: Evidence on the Fiscal Multiplier from a Quasi-Experiment. American Economic Review. 2014;104(7):2185–209. doi: 10.1257/aer.104.7.2185

[58] Xue L, Ouyang Z. Research on the influence of technology integration of digital and real industries on labor income share of enterprises. Contemp Finance Econ. 2025;:1–14. doi: 10.13676/j.cnki.cn36-1030/f.20250723.001

[59] Chen T, Tao Y. Big Data Tax Collection and Management and ESG Performance of Enterprises. Econ Rev J. 2025;2025:114–28. doi: 10.16528/j.cnki.22-1054/f.202507114

[60] Zhang R, Zhang W, Yin L. Can competition policy promote cross-regional capital flows?—Quasi-natural experiment based on the fair competition review system. Res Econ Manag. 2025;46:44–61. doi: 10.13502/j.cnki.issn1000-7636.2025.06.003

[61] Xiaoming W, Xin W, Yuehuan M, Weidong Z, Shiming L. Impact of TMT faultlines on strategic change: the joint moderating effect of environmental munificence and organizational inertia. CSS. 2025;40(4):155–68. doi: 10.3724/1005-0566.20250413

[62] Wan G, Dawod AY. ESG Rating and Northbound Capital Shareholding Preferences: Evidence from China. Sustainability. 2022;14(15):9152. doi: 10.3390/su14159152

[63] Xu C, Zhang H, Wang M, Iqbal A. Investigating the Relationship Between Entity Financialization, Managers’ Incentives, and Enterprise’s Innovation: Fresh Evidence From China. Front Psychol. 2022;12:810294. doi: 10.3389/fpsyg.2021.810294 35308072 PMC8931463

[64] Yan X, Chen X, Lu C, Liu W, Shi X, Gong Y. Strategy for sustainable supply chain transformation: a resource orchestration perspective. IMDS. 2024;124(8):2633–62. doi: 10.1108/imds-11-2023-0877

[65] Li Y, Zhang Y, Hu J, Wang Z. Insight into the nexus between intellectual property pledge financing and enterprise innovation: A systematic analysis with multidimensional perspectives. International Review of Economics & Finance. 2024;93:700–19. doi: 10.1016/j.iref.2024.03.050

[66] Wang Y, Suo L, Li C, Wang L. Cultural distance, innovation capability, and corporate performance. Finance Research Letters. 2025;77:107060. doi: 10.1016/j.frl.2025.107060

[67] Feng K, Bao C. The Impact of Environmental Management Capabilities on the Economic Value Added of Industrial Enterprises—Empirical Evidence from China. Sustainability. 2024;16(8):3356. doi: 10.3390/su16083356

[68] Sun C, Chen J. Can the Environmental Credit Evaluation Policy Alleviate the Overcapacity of Heavily Polluting Enterprises?. China and World Economy. 2025;33(1):162–94. doi: 10.1111/cwe.12570

[69] Ding M, Suardi S. Government ownership and stock liquidity: Evidence from China. Emerging Markets Review. 2019;40:100625. doi: 10.1016/j.ememar.2019.100625

[70] Lin C, Lin P, Song FM, Li C. Managerial incentives, CEO characteristics and corporate innovation in China’s private sector. Journal of Comparative Economics. 2011;39(2):176–90. doi: 10.1016/j.jce.2009.12.001

[71] Cai X, Zhu B, Zhang H, Li L, Xie M. Can direct environmental regulation promote green technology innovation in heavily polluting industries? Evidence from Chinese listed companies. Sci Total Environ. 2020;746:140810. doi: 10.1016/j.scitotenv.2020.140810 32750583

[72] Chen Y, Zhang T, Ostic D. Research on the Green Technology Innovation Cultivation Path of Manufacturing Enterprises Under the Regulation of Environmental Protection Tax Law in China. Front Environ Sci. 2022;10. doi: 10.3389/fenvs.2022.874865

[73] Jiang Y, Zhang X, Yao S. On ESG and corporate employment decision: Evidence from Chinese listed firms in 2009–2022. Economic Analysis and Policy. 2025;85:854–69. doi: 10.1016/j.eap.2025.01.004

[74] Yue W. Supply chain digitalization and corporate green innovation. Finance Research Letters. 2025;74:106656. doi: 10.1016/j.frl.2024.106656

[75] Zhao M, Fong KY, Leong WN, Li Q, Chen R. Nonexecutive employee compensation and firm labor productivity. Finance Research Letters. 2025;73:106678. doi: 10.1016/j.frl.2024.106678

[76] Sigalas C. Resource allocation choices in asset‐intensive industries. European Management Review. 2024. doi: 10.1111/emre.12697

[77] Moin CJ, Iqbal M, Malek ABMA, Khan MMA, Haque R. Prioritization of Environmental Uncertainty and Manufacturing Flexibility for Labor-Intensive Industry: A Case Study on Ready-Made Garment Industries in Bangladesh. Systems. 2022;10(3):67. doi: 10.3390/systems10030067

[78] March JG. Exploration and Exploitation in Organizational Learning. Organization Science. 1991;2(1):71–87. doi: 10.1287/orsc.2.1.71

[79] Steinberg PJ, Asad S, Lijzenga G. Narcissistic CEOs’ dilemma: The trade‐off between exploration and exploitation and the moderating role of performance feedback. J of Product Innov Manag. 2022;39(6):773–96. doi: 10.1111/jpim.12644

[80] Peng Q, Sivadasan P, Zhang R-Z. Executive compensation horizon incentives, performance targets, and auditor risk assessment. Journal of Accounting and Public Policy. 2023;42(6):107121. doi: 10.1016/j.jaccpubpol.2023.107121

[81] Zheng W, Shen R, Zhong W, Lu J. CEO Values, Firm Long-Term Orientation, and Firm Innovation: Evidence from Chinese Manufacturing Firms. Manag Organ Rev. 2020;16(1):69–106. doi: 10.1017/mor.2019.43

[82] Guan J, Liu N. Exploitative and exploratory innovations in knowledge network and collaboration network: A patent analysis in the technological field of nano-energy. Research Policy. 2016;45(1):97–112. doi: 10.1016/j.respol.2015.08.002

